# Sex-differences in catecholamine transporter expression in the rodent prefrontal cortex following repetitive mild traumatic brain injury and methylphenidate treatment

**DOI:** 10.1093/ijnp/pyaf070

**Published:** 2025-09-22

**Authors:** Eleni Papadopoulos, Anna Abrimian, Christopher P Knapp, Jessica A Loweth, Barry D Waterhouse, Rachel L Navarra

**Affiliations:** Department of Neuroscience, Rowan-Virtua School of Osteopathic Medicine, Stratford, NJ, United States; Rowan-Virtua School of Translational Biomedical Engineering and Sciences, Stratford, NJ, United States; Department of Neuroscience, Rowan-Virtua School of Osteopathic Medicine, Stratford, NJ, United States; Department of Neuroscience, Rowan-Virtua School of Osteopathic Medicine, Stratford, NJ, United States; Rowan-Virtua School of Translational Biomedical Engineering and Sciences, Stratford, NJ, United States; Department of Neuroscience, Rowan-Virtua School of Osteopathic Medicine, Stratford, NJ, United States; Rowan-Virtua School of Translational Biomedical Engineering and Sciences, Stratford, NJ, United States; Department of Neuroscience, Rowan-Virtua School of Osteopathic Medicine, Stratford, NJ, United States; Rowan-Virtua School of Translational Biomedical Engineering and Sciences, Stratford, NJ, United States; Department of Neuroscience, Rowan-Virtua School of Osteopathic Medicine, Stratford, NJ, United States; Rowan-Virtua School of Translational Biomedical Engineering and Sciences, Stratford, NJ, United States

**Keywords:** psychostimulant, VMAT2, NET, concussion, cognitive dysfunction

## Abstract

Irregular transmitter activity is theorized to underly impaired prefrontal cortex (PFC)-mediated executive functions following repetitive mild traumatic brain injury (rmTBI). The psychostimulant, methylphenidate (MPH), enhances catecholamine neurotransmission by blocking reuptake transporters and is used off-label to treat post-TBI executive dysfunction. Both rmTBI and MPH are known to independently alter catecholamine transporter levels. The present report evaluated the interactive effects of rmTBI and a sub-chronic therapeutic dose of MPH on expression levels of vesicular monoamine transporter-2 (VMAT2) and norepinephrine reuptake transporter (NET) within the medial, anterior cingulate, and orbitofrontal subregions of the PFC in both male and female rats. MPH failed to rescue, and in some cases exacerbated, rmTBI-induced reductions in VMAT2 and NET expression in males, whereas transporter expression was largely unaltered in females. These results suggest MPH treatment produces further protein-level perturbations of catecholaminergic activity that are proposed to underlie executive dysfunction in males, but negligible effects in females, following rmTBI.

## INTRODUCTION

Traumatic brain injury (TBI) often impairs prefrontal cortex (PFC)-mediated executive functions, such as attention, working memory, decision making, cognitive flexibility, and inhibitory control over behavior.^1^ The PFC contains multiple interconnected subregions: the medial prefrontal cortex (mPFC), anterior cingulate cortex (ACC), and orbitofrontal cortex (OFC), which work together to facilitate these processes. Catecholamines, dopamine (DA) and norepinephrine (NE), modulate neural activity within these subregions and thus influence their operations.^2^ Catecholamine concentrations are dysregulated following TBI,^3^^,^^4^ suggesting that these imbalances within the PFC may underlie TBI-induced executive dysfunction.

Regulatory proteins, such as those responsible for synthesis, packaging, and clearance of these transmitters, control available catecholamine concentrations. Alterations in these proteins are reported following moderate–severe TBI.^5^^,^^6^ However, mild TBI (mTBI) is the most common form of head injury, and high-risk populations, such as athletes and military personnel, are more susceptible to experiencing repeated mild TBIs (rmTBIs)^7^^,^^8^ with women often reportedly suffering worse neurological outcomes.^9^ Until recently, catecholamine regulatory proteins had not been evaluated following mild or repetitive TBI. With this gap in mind, we demonstrated that alterations in PFC catecholamine regulatory proteins are a plausible mechanism to explain sex differences in risky decision making following rmTBI.^10^^,^^11^

Psychostimulant drugs, such as methylphenidate (MPH), block catecholamine reuptake transporters and elevate transmitter concentrations throughout the brain.^12^ MPH is often prescribed off-label for TBI due to its efficacy in treating similar symptoms in patients with attention deficit hyperactivity disorder (ADHD), which also arise from catecholamine dysregulation within the PFC.^13^ At low therapeutic doses, MPH modifies catecholamine activity with regional specificity for the PFC, which limits side effects driven by subcortical elevations in catecholamine concentrations, such as hyperactivity.^14^ MPH’s reported efficacy in treating post-TBI symptoms in clinical and preclinical studies is inconsistent due to limited comprehensive investigations regarding dosage, timing, and duration of therapy, and timing and severity of the injuries, especially those categorized as rmTBIs. Additionally, sex differences in response to drug treatment and injury outcomes are not well established.^7^^,^^15^

Interestingly, chronic administration of low-dose MPH alone increases protein expression levels of regulatory transporters; vesicular monoamine transporter-2 (VMAT2), which sequesters catecholamines into presynaptic vesicles for subsequent release, and norepinephrine reuptake transporter (NET), which clears released catecholamines from the synaptic cleft through reuptake back into the presynaptic neuron, in normal functioning male rodents.^16^ However, no reports have assessed potential interactive effects of MPH and injury, or sex, on PFC transporter expression levels. Therefore, we evaluated how rmTBI and low-dose sub-chronic MPH treatment affect transporter expression levels within the mPFC, ACC, and OFC subregions of the PFC in both male and female rodents.

## METHODS

### Animals

Long-Evans rats (Envigo, 36 male and 36 female) were single-housed in a 12 hour:12 hour reverse light cycle facility and maintained on a food-regulated diet (5 g/100 g body weight/day) with *ad libitum* water.^10^^,^^11^ Animals received sham or impact surgeries in young adulthood, that is, 9-10 weeks/old. Groups for each sex (male, female) included all combinations of injury (sham, rmTBI) and treatment (saline, MPH (2 mg/kg)), totaling 8 groups. All procedures adhered to ethical considerations in accordance with the Rowan-Virtua School of Osteopathic Medicine Institutional Animal Care and Use Committee and the National Institutes of Health Guide for the Care and Use of Laboratory Animals.

### Surgeries

The closed head-controlled cortical impact (CH-CCI; Custom Design & Fabrication Inc.) model mimics, in rodents, the functional and biochemical changes of clinical mild TBI cases.^10^^,^^11^^,^^17^ CH-CCI was used to induce rmTBI (3 mTBIs within 1 week, each separated by 2 days) as previously described.^11^ Briefly, animals were anesthetized with isoflurane and the skull was exposed by a 2 cm midline incision. A 5 mm rounded metal impactor tip was aligned with bregma and zeroed along the sagittal suture, then electronically driven at 5.5 m/s to a 3.5 mm depth below surface with 100 ms dwell time.

### Dosing

Methylphenidate hydrochloride (Sigma Aldrich) was dissolved in sterile saline and injected intraperitoneally (i.p.) in 1 mL/kg volume. Low-dose MPH (2 mg/kg) falls within the therapeutic plasma range for ADHD treatment in humans (8-40 ng/mL) and enhances cognition or reduces impulsivity in various rodent preclinical assays.^14^^,^^18–21^ The first dose was administered immediately following the first surgical preparation and continued daily at the same time for 9 days, concluding 48 hour post-final surgical preparation.

### Western Blotting

One hour post-final dose (48 hour post-final surgical preparation), animals were anesthetized, and the mPFC, ACC, and OFC^22^ were dissected to determine protein expression levels of VMAT2 (responsible for packaging catecholamines into vesicles for subsequent release at the synapse) and NET (responsible for catecholamine uptake and synaptic clearance). The dopamine transporter (DAT) also clears synaptically-released DA and is also blocked by MPH, but it is sparsely expressed within the PFC and therefore, was not evaluated in this study.^23^ As previously described,^10^^,^^11^ protein (15 μg/lane) from the collected tissue was electrophoresed, transferred to polyvinylidene difluoride membranes (Bio-Rad), and probed with rabbit anti-VMAT2 (1:1000; Abcam) or anti-NET (1:1000; Abcam) primary antibody, followed by goat anti-rabbit secondary antibody conjugated with peroxidase (1:10000; Rockland Immunochemicals, Inc.). β-actin (1:2000; MilliporeSigma) was the loading control. Blots were imaged using Azure c400 Biosystems imaging system and analyzed using AzureSpot Analysis Software (Azure Biosystems).

### Statistical Analysis

Analysis was performed using GraphPad Prism software. Data from males and females were combined and analyzed together, and then separately using an ordinary two-way analysis of variance (ANOVA) with injury and treatment conditions as between-subject factors. Dunnett’s multiple comparisons tests were used to compare individual group differences to the sham/saline control group when a main effect or interaction was found. Statistical significance was determined by a *P*-value < .05.

## RESULTS

### RmTBI and MPH Effects on VMAT2 Expression Levels

#### VMAT2 Expression Levels in the mPFC

**Figure 1:**
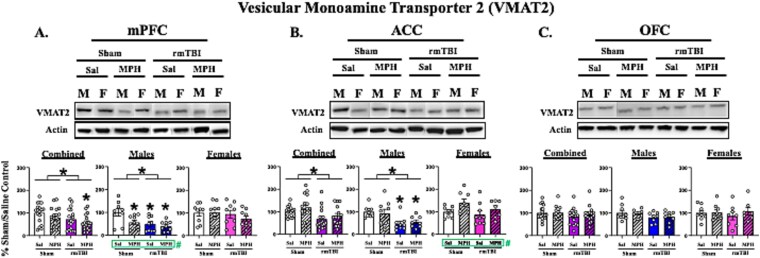
Protein expression levels of VMAT2. Left panel (A): Medial prefrontal cortex (mPFC) subregion. Middle panel (B): Anterior cingulate cortex (ACC) subregion. Right panel (C): Orbitofrontal cortex (OFC) region. Graphs represent mean percent change in total protein levels ± SEM as compared to sham/saline controls 48 hours post-final surgery and 1-hour post-final dosing for both sexes combined, males, and females. *n* = 6-9 per sex per experimental group. Main effects of injury were demonstrated when males and females were combined and in males alone within the mPFC and ACC subregions. Main effects of treatment were demonstrated in males alone within the mPFC but in females alone within the ACC. As compared to sham/saline, VMAT2 expression levels were reduced in male sham/MPH, rmTBI/saline, and rmTBI/MPH groups within the mPFC and in male rmTBI/saline and rmTBI/MPH groups within the ACC. No differences were found in the OFC. * over brackets denotes a main effect of injury and # denotes a main effect of treatment *P* < .05 following analysis with two-way ANOVA. * denotes *P* < .05 from sham/saline analyzed with two-way ANOVA and Dunnett’s multiple comparisons tests. Representative bands for the specific experimental group were cut to align the bands with the appropriate graph. Abbreviations: VMAT2 (vesicular monoamine transporter-2), mPFC (medial prefrontal cortex), ACC (anterior cingulate cortex), OFC (orbitofrontal cortex), rmTBI (repetitive mild traumatic brain injury), Sal (saline), MPH (methylphenidate; 2 mg/kg), M (male), and F (female).

Analysis of VMAT2 protein expression levels within the mPFC when both sexes were combined revealed a main effect of injury [F (1, 68) = 6.210; *P* = .0151] (Figure 1A). Although VMAT2 showed reductions in both sham/MPH and rmTBI/saline groups, only the combination of rmTBI/MPH resulted in significant VMAT2 reduction as compared to sham/saline controls (*P* = .0097) in Dunnett’s multiple comparisons tests. When animals were analyzed separately by sex, a main effect of injury [F (1, 32) = 8.450; *P* = .0066] and a main effect of treatment [F (1, 32) = 5.148; *P* = .0301] were observed in male animals. Multiple comparisons revealed a reduction in VMAT2 levels in sham/MPH (*P* = .0285), rmTBI/saline (*P* = .0094), and rmTBI/MPH (*P* = .0025) male groups. However, no significant differences were found in females [all Fs (1, 32) > 0.04549; and ps > 0.4460] (Figure 1A). These results demonstrate that injury-induced decreases in mPFC VMAT2 are primarily driven by males, and suggest these reductions following both rmTBI and MPH alone are further exacerbated when both manipulations were combined.

#### VMAT2 Expression Levels in the ACC

Analysis of VMAT2 protein expression levels within the ACC of both sexes combined revealed a main effect of injury [F (1, 63) = 9.484; *P* = .0031] (Figure 1B), with no group differences found in the post-hoc analysis. When animals were analyzed separated by sex, a main effect of injury was found in males [F (1, 31) = 13.48; *P* = .0009] (Figure 1B) with specific reductions in VMAT2 levels within the rmTBI males treated with saline (*P* = .0114) and MPH (2 mg/kg) (*P* = .0438), suggesting that MPH treatment does not rescue rmTBI-induced decreases in VMAT2 expression. A main effect of treatment was found in females [F (1, 28) = 4.575; *P* = .0413], with no observed experimental group differences (Figure 1B).

#### VMAT2 Expression Levels in the OFC

Analysis of VMAT2 protein expression levels within the OFC revealed no significant differences when sexes were combined [all Fs (1, 60) > 1.921; and ps > 0.1708] (Figure 1C), nor when males [all Fs (1, 28) > 3.635; and ps > 0.0669] and females [all Fs (1, 28) > 0.06579; and ps > 0.7994] (Figure 1C) were analyzed separately. These results suggest that VMAT2 expression is not altered by the specific rmTBI parameters used in the current experiments, treatment with 2 mg/kg of MPH, nor the combination within the OFC subregion.

### RmTBI and MPH Effects on NET Expression Levels

#### NET Expression Levels in the mPFC

**Figure 2:**
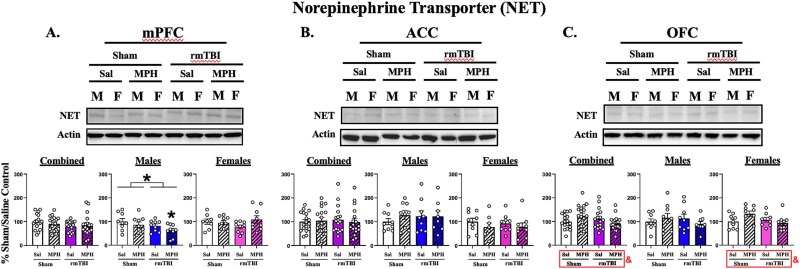
Protein expression levels of NET*.* Left panel (A): Medial prefrontal cortex (mPFC) subregion. Middle panel (B): Anterior cingulate cortex (ACC) subregion. Right panel (C): Orbitofrontal cortex (OFC) region. Graphs represent mean percent change in total protein levels ± SEM as compared to sham/saline controls 48 hours post-final surgery and 1-hour post-final dosing for both sexes combined, males, and females. *n* = 8-9 per sex per experimental group. A main effect of injury was demonstrated in males alone within the mPFC. An injury-by-treatment interaction was demonstrated when males and females were combined and in females alone within the OFC. As compared to sham/saline, NET expression levels were reduced in the male rmTBI/MPH group within the mPFC. No differences were found in the ACC. * over brackets on graph denotes a main effect of injury and & denotes an injury-by-treatment interaction *P* < .05 following analysis with two-way ANOVA. * denotes *P* < .05 from sham/saline analyzed with two-way ANOVA and Dunnett’s multiple comparisons tests. Representative bands for the specific experimental group were cut to align the bands with the appropriate graph. Abbreviations: NET (norepinephrine reuptake transporter), mPFC (medial prefrontal cortex), ACC (anterior cingulate cortex), OFC (orbitofrontal cortex), rmTBI (repetitive mild traumatic brain injury), Sal (saline), MPH (methylphenidate; 2 mg/kg), M (male), and F (female).

Analysis of NET protein expression levels within the mPFC when both sexes were combined revealed no significant differences [all Fs (1, 67) > 0.04458; and ps > 0.1116] (Figure 2A). When separated by sex, no significant differences were found in females [all Fs (1, 31) > 0.1421; and ps > 0.1139], yet a main of effect of injury was found in males [F (1, 32) = 4.365; *P* = .0447]. Dunnett’s multiple comparisons revealed a significant reduction in mPFC NET expression levels within the rmTBI/MPH group only (*P* = .0343), suggesting that this combination of injury and treatment with 2 mg/kg of MPH is necessary to reduce NET expression within the mPFC of male animals.

#### NET Expression Levels in the ACC

Analysis of NET protein expression levels within the ACC of both sexes combined revealed no significant differences [all Fs (1, 66) > 0.01304; and ps > 0.6164] (Figure 2B). When separated by sex, no significant differences were found in males [all Fs (1, 31) > 0.1978; and ps > 0.4726] nor females [all Fs (1, 31) > 0.03892; and ps > 0.2285] (Figure 2B). These results suggest that NET expression is not altered by injury, MPH treatment, or the combination of these factors within the ACC subregion.

#### NET Expression Levels in the OFC

Analysis of NET protein expression levels within the OFC of both sexes combined revealed an injury-by-treatment interaction [F (1, 67) = 5.492; *P* = .0221], with no group differences in the post-hoc analysis (Figure 2C). When animals were analyzed, separated by sex, no significant differences were found in male animals [all Fs (1, 32) > 0.06091; and ps > 0.2028] (Figure 2C). However, an injury-by-treatment interaction was found in females [F (1, 31) = 4.502; *P* = .0420]. This result suggests a female-driven sex-dependent effect within this subregion, yet no experimental group differences were found in the post-hoc analysis (Figure 2C).

## DISCUSSION

The present results demonstrate rmTBI and low-dose MPH display interactive effects on catecholamine transporter expression within the PFC in a sex-dependent manner. RmTBI alone reduced transporter expression in specific subregions. These effects were primarily driven by males, with more pronounced effects in those who received MPH. Meanwhile, transporter expression in females was mainly unaltered. Additionally, this is the first report to show alterations in PFC VMAT2 expression following a closed-head rodent model of rmTBI.

In the mPFC and ACC, rmTBI reduced expression levels of VMAT2 when both sexes were analyzed together. However, when analyzed separately, these findings were significant in males only (Figure 1A & B). NET expression was also selectively decreased in the mPFC following injury in males (Figure 1A). Together, decreased transporter levels following rmTBI suggest a compensatory response to low catecholamine levels following TBI, as previously reported.^3^ In an injury-induced hypo-catecholaminergic state, decreased transmitter concentrations signal less substrate availability and therefore, less need for reuptake and repackaging following injury, which leads to downregulation of the transporters required to mediate these actions.^24^ We have previously reported decreases in OFC NET expression in both males and females following a milder rmTBI protocol (impact depth = 2.5 mm^10^), but no change in overall PFC NET expression using the current parameters.^11^ We suggested these rmTBI parameters may be disrupting feedback sensitivity to behavioral outcomes and altered catecholamine activity, thereby preventing a compensatory response. While an overall injury effect was detected within the mPFC of males here, it is clear this effect was driven by those who were treated with MPH. We also acknowledge the nature of investigating mild forms of TBI inherently yields more subtle and less consistent results as compared those of more severe TBI. Nonetheless, the present results corroborate with the latter report in which we infer these parameters of rmTBI prevent compensation of catecholamine disturbance following rmTBI alone.

MPH treatment alone decreased VMAT2 levels within the mPFC of uninjured males (Figure 1A). Although there was a main effect of treatment on VMAT2 expression within the ACC of females, there was no directional trend for any experimental group (Figure 1B). No significant changes in NET expression were observed with MPH alone (Figure 2). Low-dose MPH is known to inhibit the NET transporter within the PFC,^14^ which decreases reuptake of catecholamines. Based on evidence provided by the current study, it is plausible that lasting decreases in intracellular catecholamine levels would signal decreased need for repackaging and reduced VMAT2 expression in these uninjured MPH-treated males.

The findings in the present study contrast with another report demonstrating increased VMAT2 and NET expression levels within the frontal cortex of uninjured male rodents using a similar MPH regimen.^16^ The main difference being that the entire frontal cortex was analyzed, whilst here, we focused on specific PFC regions pertaining to executive functions that govern decision-making behaviors, which may explain the contrasting transporter findings. By using localized subregions, this allows for a more targeted approach to understanding catecholamine regulation to specific PFC-mediated behaviors rather than a global view of overall PFC function and alterations. Nevertheless, we revealed sex differences in biochemical response to low-dose MPH treatment in normal functioning rodent PFC sub-regions.

Finally, the main goal of this study was to determine the interaction between rmTBI and low-dose MPH treatment on transporter expression in male and female rodents. The rmTBI-induced decrease in VMAT2 levels in males persisted following MPH treatment within the mPFC and ACC (Figure 1A & B). Additionally, only the combination of rmTBI and MPH was sufficient to significantly reduce NET expression levels within the mPFC of males as compared to their sham/saline controls (Figure 2A). In this study, we hypothesized that when NET was blocked with MPH, the catecholamine molecules that remained following rmTBI would persist within the synapse and mitigate the injury-induced hypo-catecholamine state. This increase in extracellular substrate availability would then signal the requirement for reuptake and repackaging, resulting in upregulation of NET and VMAT2 to normal pre-rmTBI levels. Quansah and Zetterström’s (2019) report demonstrating increased VMAT2 and NET expression levels within the PFC following MPH treatment further supported these expectations. However, we did not observe the proposed MPH-induced normalization of transporter levels following rmTBI. It is possible that the catecholamine state within the PFC of males following rmTBI may either be unsalvageable using this dosing paradigm and/or at this time point. Although MPH was administered daily, catecholamine elevations within the PFC returned to baseline levels within 3 hours following each dose.^14^ These acute bouts, rather than persistent elevation, could further aggravate injury-induced neuroplastic changes to homeostatic mechanisms regulating transporter expression that result in further downregulation, rather than restoration. Nonetheless, we reveal the potential for MPH to exacerbate rmTBI-induced biochemical signatures that may underlie further impairments of executive function in males, especially during this critical time point of young adult PFC development.

Interestingly, we observed overall treatment and injury-by-treatment effects on transporter levels following rmTBI in females, but no effects of injury alone or specific directional trends for any of the experimental groups. The sex-specific effects induced by MPH on transporter expression following injury could be due to differential pharmacokinetics and circulating drug concentrations in males vs females.^25^ For example, sex-specific MPH-induced influences on performance of behavioral tasks and drug sensitivity have been reported, where a higher MPH dosing regimen improved injury-induced spatial memory deficits in males but increased locomotor activity in females, respectively.^15^ Indeed, the observed sex differences could reflect the mere fact that the female brain is under constant hormonal flux,^26^ providing an additional layer of influence over responses to rmTBI and drug-induced alterations of transmitter concentrations and protein expression levels.

Overall, the present report demonstrates that regulation of catecholamine transporter expression within the PFC is dynamic and adaptive in response to experimental injury and drug manipulations that perturb catecholamine homeostasis. These results are particularly timely given MPH is commonly used off-label in clinical settings to treat post-TBI executive dysfunction. Further investigation is required to pinpoint alterations in protein functionality (ie, phosphorylation, epigenetic modifications, or mislocalization, etc.), as these may also contribute to catecholamine imbalance. Although these interactive effects on transporter expression are a glimpse into the catecholaminergic regulatory processes, the present report suggests that this dosing regimen of MPH exacerbates protein-level perturbations in males following rmTBI, but has negligible effects in females. Future directions will use this experimental design to investigate other key proteins involved in catecholamine regulation and behavioral outcomes following therapeutic doses of MPH and additional pharmacotherapies that target the catecholaminergic systems following rmTBI. To conclude, this work highlights the importance of considering sex-dependent treatment strategies associated with off-label use of MPH for treating post-TBI executive dysfunction.

## Data Availability

The data underlying this article will be shared on reasonable request to the corresponding author.
